# Advances in *in vivo* magnetic resonance spectroscopy for metabolic disorders

**DOI:** 10.3389/fendo.2025.1578333

**Published:** 2025-07-18

**Authors:** Yuliya Kupriyanova, Vera Schrauwen-Hinderling

**Affiliations:** ^1^ Institute for Clinical Diabetology, German Diabetes Center, Leibniz Center for Diabetes Research at Heinrich Heine University Düsseldorf, Düsseldorf, Germany; ^2^ German Center for Diabetes Research (DZD), Partner Düsseldorf, Neuherberg, Germany; ^3^ Department of Radiology and Nuclear Medicine, Maastricht University Medical Center, Maastricht, Netherlands

**Keywords:** magnetic resonance spectroscopy, metabolic health, diabetes, lipid metabolism, glycogen, mitochondrial function

## Abstract

Magnetic Resonance Spectroscopy (MRS) and Magnetic Resonance Imaging (MRI) yield valuable metabolic information in a non-invasive way. The current mini review addresses current practice and recent advances in metabolic research, specifically in the field of obesity, insulin resistance and diabetes. The potential application of MRS to investigate lipid and glycogen stores, as well as energy metabolism are reviewed and novel methods to extract more detailed information on fatty acid composition or newly detectable metabolites such as acetylcarnitine or nicotinamide adenosine dinucleotide (NAD^+^) and NADH are discussed. These advances are based on optimization of post-processing or on the application of new schemes for spectral editing to suppress unwanted signal. The advantage of MRS is that it gives real-time dynamic information and therefore, metabolism can be investigated during physiological challenges, such as exercise, food intake or immediate drug action. Due to its non-invasive nature, repeated measurements are possible with MRS, to monitor treatments and interventions and also organs that are not easily accessible for biopsies, such as the liver or the heart can be probed by MRS. When investing in further methodological development, new applications will arise, advancing our understanding of metabolic disease and giving us tools to identify successful treatment and prevention strategies in individuals at risk.

## 1 Introduction

Magnetic Resonance Spectroscopy (MRS) is a non-invasive method to investigate metabolism *in vivo* and has proven to be very valuable for research of metabolic disorders (1, 2). MRS is based on the same physical principle as magnetic resonance imaging (MRI), but instead of anatomical information, MRS provides biochemical information about the measured tissue and quantifies distinct metabolites *in vivo*. Combining MRI and MRS, i.e. acquire an MR image first as a reference for further positioning the region of interest for MRS acquisition, allows one to probe metabolism in a specific location, which is of primary interest for clinical and preclinical studies using MRS. Information on the basic principles of MRS can be found in text books, such as (3). Recently published experts’ consensus recommendations overview terminology and concepts as well as reporting standards for *in vivo* MRS research (4, 5). An MRS signal can be acquired by interaction with a feature of the nucleus, which is called nuclear spin. Atomic nuclei of several- but not all- elements have spin (where spin ≠ 0) and are thus detectable by MRS. In metabolic research, signal acquisition from 1H, 13C and 31P isotopes is most often used and recently also 2H (6–9). 1H is very abundant constituent of water, lipids and carbohydrates and the sensitivity for MRS is relatively high, making it often the method of choice to observe such metabolites. MRS using other nuclei than 1H (eg 31P, 13C and 2H), are termed multinuclear, or x-nuclear methods.

13C is a stable isotope of carbon with a spin of ½. This is in contrast to the isotope 12C which is MRS invisible. Most of the naturally abundant carbon represents 12C, and 13C has a low natural abundance of only 1.1%. Furthermore, the sensitivity for 13C-MRS is low, due to a low gyromagnetic ratio (four times lower than 1H), which leads to its low detection efficiency. Nevertheless, 13C-MRS is usually the method of choice when quantifying glycogen and regardless of the fact that only 1.1% contributes to the glycogen signal that can be detected, this natural abundance of 13C is enough to generate a measurable signal. The low natural abundance of 13C makes it an interesting candidate for tracer studies to study metabolic fluxes. Interestingly, the recent ‘rediscovery’ of deuterium MRS, which also has a low natural abundance, but requires less sophisticated MRS sequences for detection (6), has opened alternative possibilities to perform *in vivo* tracer studies by x-nuclei MRS. 31P has a spin of 1/2 and its natural abundance is high (approximately 100%), however due to a lower gyromagnetic ratio, its sensitivity is 2,5 times lower than for 1H. 31P-MRS is used to get insights into energy metabolism, as this method can be employed to quantify high energy metabolites such as adenosine triphosphate (ATP) or creatine phosphate (PCr).

**Figure 1:**
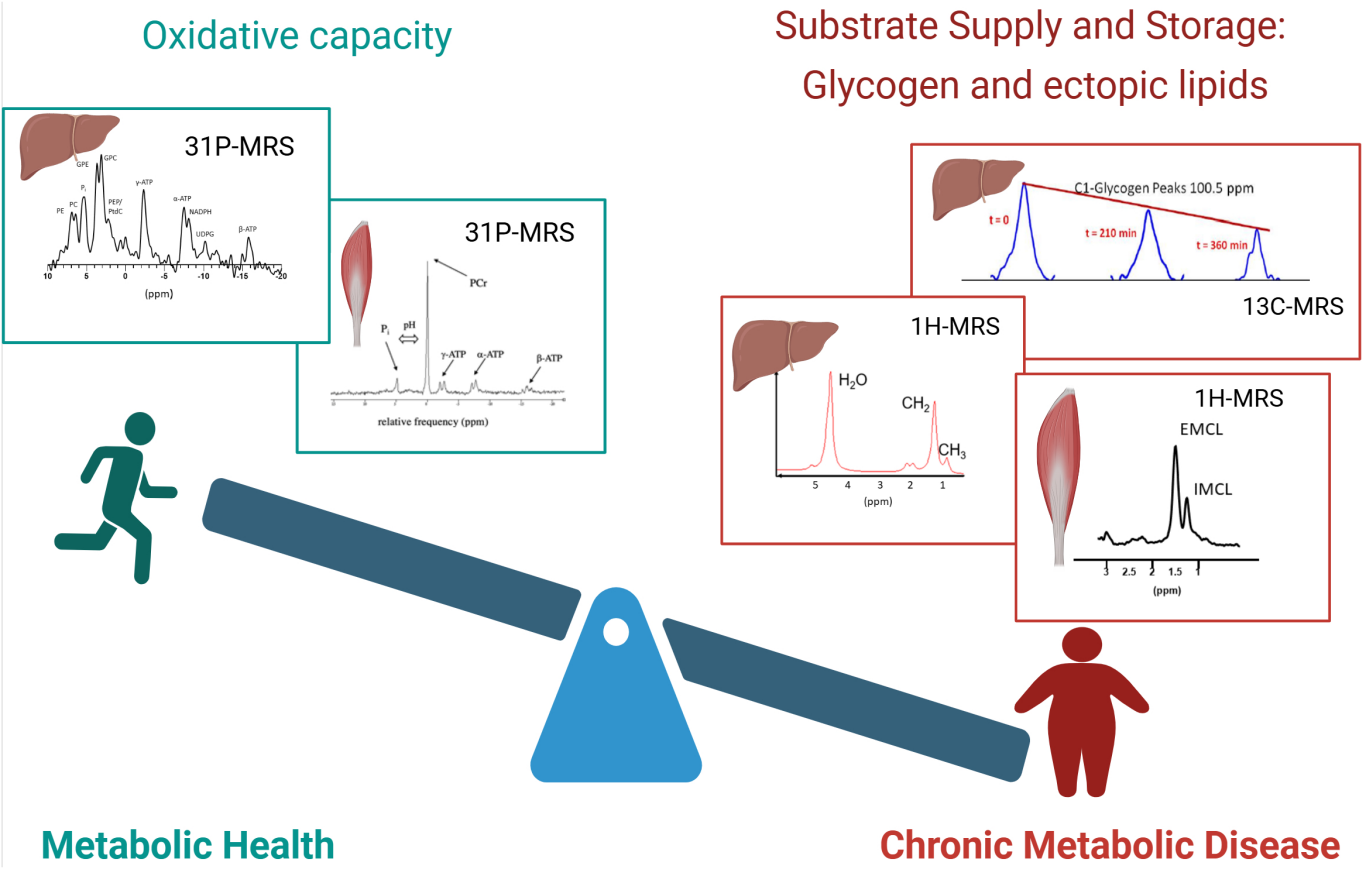
Balance of metabolic health. The balance between substrate availability and the capacity to handle the substrate load is a strong determinant of metabolic health. Left: metabolically healthy people are characterized by a high oxidative capacity, which can be determined by organ-specific 31P-MRS. Examples of 31P spectra of the liver and muscle are shown. Right: people with chronic metabolic disease are generally characterized by increased lipid content and disturbed glycogen dynamics, which can be detected by 1H-MRS and 13C-MRS respectively. Top right: Sequential 13C spectra of glycogen are shown, which enable the determination of net glycogen flux. Bottom right: Examples of 1H spectra of the liver and muscle are shown. Created in BioRender. (Kupriyanova, Y. (2025) https://BioRender.com/dalrhjx).

For metabolic health, the balance between substrate availability and the capacity to handle the substrate load is crucial and many metabolic diseases, such as type 2 diabetes are strongly associated with obesity and a lack of physical activity (**Figure 1**). A chronically, and constantly high supply of fatty acids and carbohydrate to muscle tissue and the liver are typical for these conditions. This leads to expansion of substrate stores. At the same time, chronic metabolic disease was also associated with hampered oxidative capacity, leading to a metabolic ‘gridlock’ (10). Such imbalance of substrate supply and oxidative capacity is typical for chronic metabolic diseases like liver steatosis and metabolic steatohepatitis (MASH) (11), insulin resistance and type 2 diabetes (12). It was reported that the combination of low oxidative capacity and high substrate availability leads to accumulation of metabolic intermediates, such as acyl-CoA, ceramides and diacylglycerols (DAG), which were implicated in the etiology of insulin resistance (13). Information on both sides of this balance (substrate stores and oxidative capacity) can be gained by MRS and can yield important insights to better understand metabolic health and disease.

The aim of the current review is to represent the most recent advances in *in vivo* MRS for research of metabolic disorders, discuss recent applications of innovative MRS methods and provide ideas for the future directions of MRS in metabolic research.

## 2 Lipid and glycogen storage in organs

### 2.1 1H-MRS for assessment of ectopic lipid depots

Most surplus energy is stored in the form of triglycerides in adipose tissue. However, fatty acids can also be stored outside adipose tissue as triglycerides in lipid droplets, in muscle, liver and the myocardium. This especially occurs in overweight and obesity. Adipose tissue around organs (visceral adipose tissue) is also often considered ‘ectopic’, as expansion of these adipose tissue stores were associated with an unhealthy phenotype. The expansion of ectopic lipid stores were strongly associated with insulin resistance (14–16), which is an early hallmark of the type 2 diabetes development.

While MRI can best be used to quantify the volume of various adipose tissue depots on whole body level (eg subcutaneous and various kinds of visceral adipose tissue depots) (17), 1H-MRS is the method of choice for monitoring ectopic fat depots in the muscle, liver and myocardium (18, 19). In skeletal muscle, the differentiation of intramyocelleluar lipids (IMCL), representing lipid droplets in muscle cells and lipids from intramuscular adipose tissue (extramyocellular lipids, EMCL) is possible with 1H-MRS (20, 21). These measurements are mostly performed in the tibialis anterior or vastus intermedius muscle, because the separation of IMCL and EMCL is best achieved in these muscles (22). In the liver and the heart, there is no infiltrating adipose tissue and proton spectra from these organs are simpler to analyse and the separation between lipid signal from parenchymal cells and lipid signal from adipose tissue is therefore not an issue (19). 1H-MRS is considered to be the gold-standard for non-invasive quantification of hepatic lipid content (23). MRS is used for diagnosing of metabolic dysfunction associated steatotic liver disease (MASLD), one of the complications of diabetes mellitus (24) and for monitoring of longitudinal changes in hepatic lipid content due to diabetes development (25).

### 2.2 Origin of ectopic lipids

Next to simply quantifying the content of ectopic lipids, MRS can provide a more detailed characterisation and importantly, can thereby provide insight into the origin of the stored lipids. Especially in the liver, there are various pathways that contribute to lipid accumulation and it is clinically important to understand what the contribution is from each of the various pathways, such as *de novo* lipogenesis, adipose tissue lipolysis and direct triglyceride storage from meals.

#### 2.2.1 *De novo* lipogenesis (DNL)

Regarding DNL, it is interesting to note that in humans, upregulated rates of DNL are expected to result in mainly saturated fatty acids (SFA) and therefore, the quantification of specifically SFA could give information about rates of *de novo* lipogenesis. By optimizing and applying a sophisticated post-processing routine, it was shown that SFA, monounsaturated fatty acids (MUFA) and polyunsaturated fatty acids (PUFA) could be specifically quantified in the liver (26). Using this method, it could indeed be shown that hepatic SFA is correlated to rates of DNL (26) and that the fraction of SFA reflects DNL.

#### 2.2.2 Meal-derived hepatic lipids

In order to quantify lipid storage from a meal, tracer experiments are needed. Fatty acids, enriched in 13C isotopes are stable (nonradioactive) molecules that can be incorporated into a meal and tracked to their storage site by MRS. Normally, 13C MRS would be the method of choice. Due to the low sensitivity and the very crude localisation of this method, indirect 13C methods are more appropriate. Such 1H-observed, 13C-edited MRS methods are selective for 13C signal, while retaining the sensitivity and localisation possibilities of 1H-MRS (also called POCE for proton-observed, carbon edited). In the liver, the most broadly used POCE approaches rely on subtraction of two consecutively acquired spectra (with/without the 13C editing pulses). In the liver, where respiratory motion is not negligible, such subtraction techniques are not successful due to motion-induced subtraction artifacts. To this end, a single shot POCE technique (based on the seletion of heteronuclear quantum coherences, HMQC) was developed and applied in a 13C fatty acid tracking experiment. The incorporation of 13C labelled fatty acids resulted in increased hepatic 13C enrichment after the meal and it was shown that this meal-derived lipid storage in the liver was similar in lean and obese volunteers (27).

#### 2.2.3 Adipose tissue derived lipids

Regarding the contribution of fatty acids originating from adipose tissue, most results in the literature originate from studies employing other techniques than MR methods, such as PET or stable isotopes in combination with biopsies (28, 29). However, also MRS results support the importance of fatty acids originating from adipose tissue, eg from studies provoking adipose tissue lipolysis and high concentrations of plasma free fatty acid (FFA), and showing that this results in increased hepatic lipid content (30).

Further increase in the sensitivity of the indirect 13C MRS methods are warranted to decrease the amount of tracer needed for the experiments and make the broader application more realistic. Currently, such experiments are very expensive. Another promising approach which has drawn the interest of the scientific community is the *in vivo* application of deuterium MRS (also called deuterium metabolic imaging, DMI), which can be combined with the consumption or infusion of deuterated substrate to observe storage pathways and metabolic fluxes (6, 9).

### 2.3 13C-MRS to determine hepatic glycogen

As mentioned above, glycogen has been quantified by 13C-MRS, using the natural abundance signal of 13C (31). In combination with other measurements, the quantification of glycogen storage and utilization has provided insight into different processes of glucose metabolism (glucogenesis, glucogenolysis, gluconeogenesis), and is useful for investigation of disturbed glycogen dynamics in diabetes as well as the effects of antidiabetic therapies (32, 33). The interest in glycogen dynamics is renewed due to the successful action of GLP-1 agonists, which (amongst others) target glycogenolysis (34).

However, the MRS-based quantification of glycogen has some limitations. The measurement time is long, and in many studies, localization was rather crude and typical values from the absolute quantification, based on phantom replacement experiments, vary greatly between sites (35). However, significant improvements have been made to apply more sophisticated localisation in 13C MRS, for example, rat studies show the feasibility of adding outer volume suppression to improve localisation (36).

Increasing 13C enrichment before the start of the measurements by ingestion/infusion of 13C enriched substrate, make the measurement more sensitive, but again, such experiments are very costly, due to the high costs of stable isotope tracers. Proton MRS was positioned as a potential alternative to 13C in glycogen quantification (37), overcoming the sensitivity issues, however, these attempts are not very robust and hindered by saturation transfer phenomena when water suppression is used (38). It was also demonstrated that specialized, multi-element coils increase the signal detection significantly and thereby help to reduce the measurement time (39). The reliability of the 13C-based phantom replacement experiments may be improved by more advanced, imaging-based signal correction, based on coil sensitivity maps that are projected on the actual experimental set-up. Further technical improvements are warranted to make it easier and faster to non-invasively monitor glycogen in the liver and muscle in order to elucidate 24 hour glycogen dynamics in health and disease and to investigate the effect of treatment.

Indirect detection of glycogen using chemical exchange saturation transfer imaging (glycoCEST) methods is a promising alternative to 13 C measurements (40).

## 3 Energy metabolism; oxidative capacity

While stimulating certain pathways of substrate storage can favour metabolic disease and challenge metabolic health, the capacity to handle substrate, the oxidative capacity, is also a strong determinant of metabolic health. Indeed, it is well known that intervention that improve oxidative capacity, such as endurance training, improve metabolic health. In skeletal muscle, a low oxidative capacity was found in persons with type 2 diabetes (41) and even in individuals who are still healthy but at risk for type 2 diabetes development (42). In the liver, low ATP concentrations were detected in type 2 diabetes (43) and during the progression from liver steatosis of liver inflammation, mitochondrial capacity was shown to be decreased (11, 44).

To investigate energy metabolism and mitochondrial function non-invasively, 31P-MRS is very valuable, as phosphorous containing metabolites, such as ATP, PCr and inorganic phosphate (Pi) can be detected and quantified. An overview of different 31P-MRS based methods for energy metabolism measurements is given in (45).

### 3.1 Energy metabolism and mitochondrial function

A well-established way of investigating oxidative metabolism in muscle is by investigating PCr recovery kinetics after PCr depletion, typically after exercise performed inside the scanner. It has been shown that PCr resynthesis is fuelled almost purely by aerobic pathways and the half-time (or rate constant) of the monoexponential PCr recovery is reflecting oxidative capacity (46). This method was first applied several decades ago and still considered as a very robust tool to investigate oxidative metabolism (47, 48).

Next to getting information about maximal *in vivo* oxidative capacity from PCr recovery, monitoring PCr at the onset of exercise is also interesting. During this phase, the prolonged dependence on PCr hydrolyses for energy supply reflects slow mitochondrial activation, also termed mitochondrial inertia (48), which was associated with exercise intolerance and metabolic disease (49). For more detailed discussion of such metabolic inertia, we refer to a review on this topic (50).

Another way to investigate energy metabolism is by applying saturation transfer measurements and quantifying (unidirectional) ATP synthetic fluxes. While this method has been initially positioned as a way to quantify mitochondrial ATP synthesis and thereby mitochondrial function (51), there was also critique on such interpretation (52). It was shown that the rates determined by saturation transfer are complex to interpret (53) and probably contain substantial part of anaerobic metabolism (52). Results relating unidirectional Pi-ATP fluxes, as assessed by saturation transfer measurements and oxidative capacity, as assessed by PCr recovery are mixed with some data showing a positive correlation between the two markers of energy metabolism (54), while a rodent study that manipulated mitochondrial function with injections of a complex I inhibitor, did not find a relationship between Pi-ATP flux and oxidative capacity (55).’

A different method to investigate aerobic metabolism is by using 13C tracer, such as 13C acetate, in combination with 13C-MRS to quantify downstream metabolites, such as glutamate (56) and thereby determine the tricarboxylic acid (TCA) cycle flux.

Another important player in mitochondrial function and especially mitochondrial biogenesis is nicotinamide adenosine dinucleotide (NAD^+^) (57). NAD^+^ activates deacetylases, which lead to deacetylation of transcription factors, such as peroxisome proliferator-activated receptor gamma coactivator 1 – alpha (PGC1-α), so that they can enter the nucleus and lead to translation of mitochondrial genes and therefore to mitochondrial biogenesis. In principle, NAD^+^ and NADH can be detected by 31P-MRS, however, a complicating factor is the overlap with ATP resonances, making it impossible to quantify NAD^+^ and NADH in muscle by direct 31P-MRS, at least at clinical field strength. Here, the development of a new sequence that selectively suppresses ATP, sparing NAD^+^ and NADH (58) can be instrumental. Using this sequence, it was shown that the decrease in NAD^+^, seen during aging is blunted by regular exercise (58).

## 4 Metabolic intermediates

When the amount of substrate supplied to organs, mainly in the form of plasma glucose and plasma FFA is constantly high, while energy needs and oxidative capacity is low, this can lead to the accumulation of metabolic intermediates, hampering metabolic function (59). Therefore, quantifying metabolic intermediates may be important to monitor metabolic health. Also the capacity to buffer accumulating intermediates may be metabolically beneficial. Such an example of a metabolite is acetylcarnitine, which is formed when acetyl-CoA concentrations are high, counteracting the accumulation of acetyl-CoA.

### 4.1 Acetylcarnitine

Acetylcarnitine is formed by the conjunction of carnitine and acetyl-CoA. While acetyl-CoA, formed in the mitochondria, cannot leave the organelle, upon conjugation with carnitine, acetylcarnitine can leave the mitochondria, keeping acetyl-CoA low. The enzyme responsible for this, carnitine acetyltransferase (CrAT) was shown to have high impact on metabolic health, as knock-out animals are glucose intolerant (60). Therefore, a high capacity to form acetylcarnitine is a reflection of good metabolic health. In principle, acetylcarnitine can be quantified by 1H-MRS, however the strong lipid signals usually cover this resonance. Suppression the lipid signal by long echo time or T1 editing was shown to uncover the acetylcarnitine resonance, making the quantification possible (61, 62).

In a cross-sectional study, it was shown that acetylcarnitine concentration correlates with insulin sensitivity and that individuals with type 2 diabetes are characterized by lower concentrations of acetylcarnitine (61). The association of low acetylcarnitine with insulin resistance and glucose intolerance and low oxidative capacity was confirmed in various muscle groups at ultra-high field (7T) (63, 64). As acetyl-CoA levels can rapidly change and therefore, also acetylcarnitine concetrations adapt rapidly to the physiological state, strict standardisation is necessary and recommendations are given in the consensus paper on 1H-MRS in muscle (22).

## 5 Conclusion and outlook: potential of MRS in metabolic research

**Figure 2:**
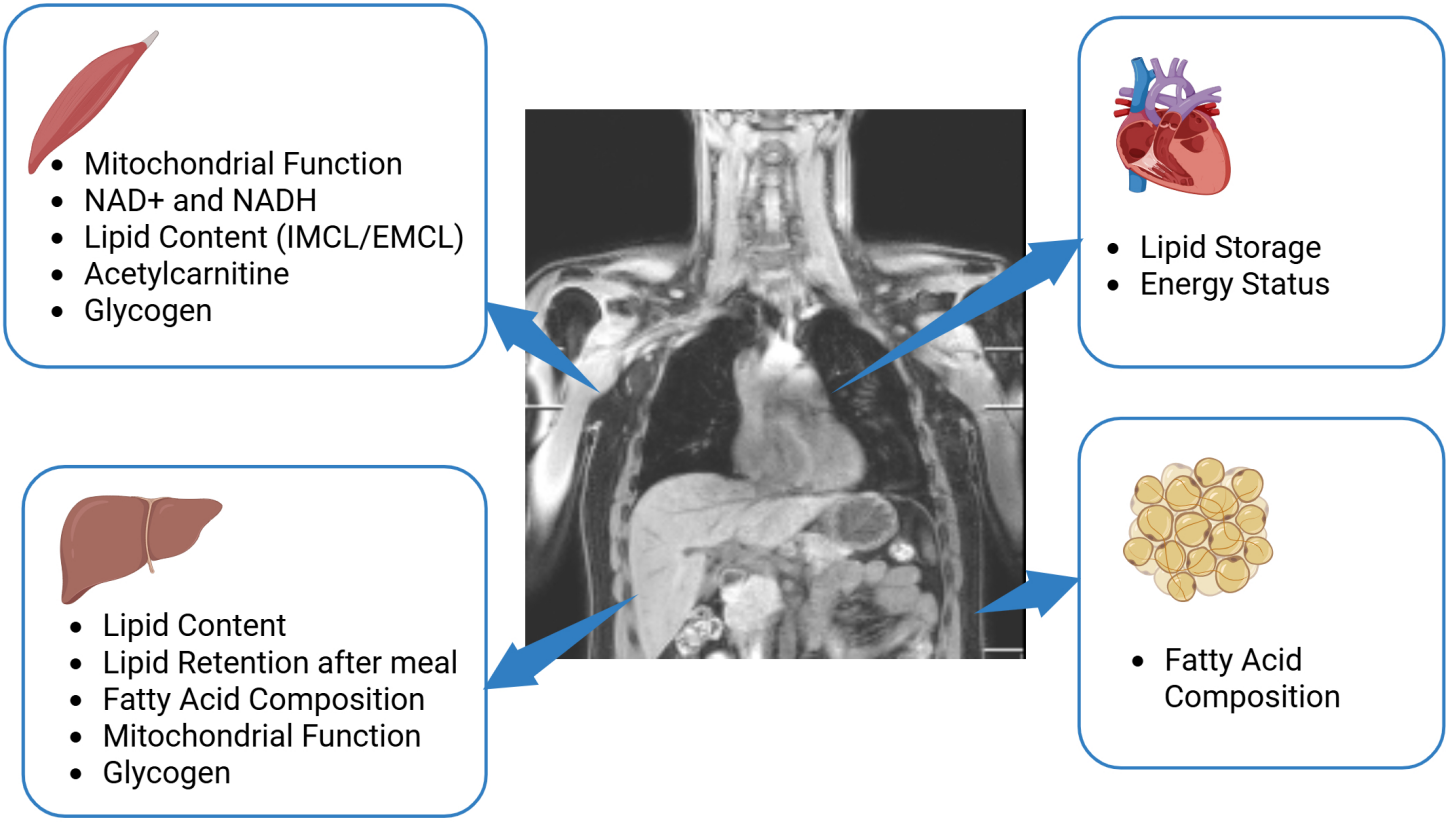
Potential of MRS in metabolic research. The application of multinuclear MRS in skeletal muscle provides valuable information about mitochondrial function, muscle lipid content, glycogen and can detect acetylcarnitine, NAD+, and NADH. The application of such techniques in the liver delivers knowledge on hepatic lipid content and lipid composition, provide information about mitochondrial function and glycogen storage and indirect 13C editing techniques enable monitoring of lipid retention after a meal. In the heart, multinuclear MRS is able to determine lipid storage and the energy status of heart by 1H-MRS and 31P-MRS respectively. In adipose tissue, 1H-MRS and 13C-MRS can provide information about fatty acid composition. Created in BioRender. (Kupriyanova, Y. (2025) https://BioRender.com/b27z305).

MRS and MRI yield invaluable metabolic information in a non-invasive way (**Figure 2**). The further methodological development is necessary to advance the field and to specifically address clinical needs. As shown by the establishment of the quantification of SFA, MUFA and PUFA, it can worth to invest into careful acquisition and post-processing, as the improved spectral quality and more accurate fitting can already uncover new, valuable information. Careful post-processing with image-based correction for coil sensitivity in glycogen measurements can also improve the robustness of 13C-MRS for glycogen detection.

Some physiologically relevant metabolites are currently not quantifiable by standard MRS due to spectral overlap with neighbouring resonances. Here, the development of spectral editing techniques to specifically suppress certain resonances, while leaving other resonances undisturbed, can be very helpful. As an example, the suppression of γ-ATP revealed the NAD+ and NADH resonances and thereby enabled the quantification of these metabolites in skeletal muscle (58).

The advantage of MRS is that it gives real-time dynamic information and therefore, metabolism can be investigated during physiological challenges, such as exercise, food intake or immediate drug action. In that respect, the combination of methods by dual, or even triple tuned coils in combination with interleaved MRS (65) opens ways to monitor various facets of metabolism at the same time, which can be very interesting to monitor various aspects of the physiological response to a challenge.

Motion is one of the crucial factors affecting quality of MRS. Experts’ consensus recommendations named and discussed three main approaches for mitigating the effect of motion in MRS (66): volunteer immobilization, retrospective correction and prospective real-time correction using various triggering and/or tracking methods (67, 68). The last can simultaneously update localization and the B_0_ field, which is essential for abdominal MRS measurements in organs affected by respiratory or cardiac motion, and prospective methods were indicated as being the method of choice in the expert consensus paper (66). A pipeline for rapid prospective motion correction of multinuclear MR Spectroscopy of the target organ using MR image navigators was recently introduced and implemented on 3T and 7T scanners. Its feasibility for reliable *in-vivo* motion extraction on the myocardium and kidney was shown in (69).

Clearly, the non-invasive nature of MRS is very valuable, as repeated measurements are possible to monitor treatments and interventions and also organs that are not easily accessible for biopsies, such as the liver or the heart can be probed by MRS. Although ultra-high field (7T) has clear advantages for MRS, most studies on metabolic health can be performed at clinical field strength (3T).
